# The effectiveness of virtual reality-based rehabilitation in patients with neck pain: a systematic review and meta-analysis with trial sequential analysis of randomized controlled trials

**DOI:** 10.3389/fpubh.2026.1933999

**Published:** 2026-10-22

**Authors:** Amu Jike, Xiang Zhang, Hao Liu, Anyun Yu

**Affiliations:** 1 First People's Hospital of Liangshan Yi Autonomous Prefecture, Xichang, Sichuan, China; 2 Department of Orthopedics, Orthopedic Research Institute, West China Hospital, Sichuan University, Chengdu, China

**Keywords:** meta-analysis, neck pain, randomized controlled trials, trial sequential analysis, virtual reality

## Abstract

**Background:**

Neck pain is a widespread condition impacting daily functioning and quality of life. Virtual Reality (VR) technology has gained attention as a non-pharmacological intervention in rehabilitative medicine, offering potential benefits for pain management. This study systematically reviewed and conducted a meta-analysis on the effectiveness of VR rehabilitation for neck pain.

**Methods:**

Following PRISMA guidelines, a comprehensive literature search was performed across databases (e.g., PubMed) up to October 1, 2024, targeting randomized controlled trials (RCTs) comparing VR-based rehabilitation with alternative interventions for neck pain. Data extraction focused on outcomes like the Visual Analogue Scale (VAS) and Neck Disability Index (NDI). Quality and bias were assessed using the Cochrane Risk of Bias Tool, and the GRADE approach evaluated evidence certainty. Trial Sequential Analysis (TSA) was also employed to ensure robustness in conclusions. This protocol was prospectively registered with PROSPERO (CRD42022334319).

**Results:**

The meta-analysis, which included 11 randomized controlled trials with 476 participants, demonstrated that virtual reality-based rehabilitation significantly reduced VAS scores [471 patients, mean difference (MD) −0.70, *p* = 0.01] and NDI scores (411 patients, MD −3.06, *p* < 0.0001) within the first week. Furthermore, significant reductions in VAS (315 patients, MD −1.00, *p* < 0.05) and NDI scores (315 patients, MD −3.81, *p* = 0.005) were observed from the first week to 6 months in the VR-based rehabilitation group. Trial sequential analysis further confirmed the statistical strength and reliability of these findings. Improvements in cervical mobility were observed, as evidenced by increased neck flexion and extension.

**Conclusion:**

VR-based rehabilitation shows promise as an effective strategy for managing neck pain, improving both pain levels and functional capacity.

**Systematic review registration:**

https://www.crd.york.ac.uk/PROSPERO/view/CRD42022334319, identifier PROSPERO (CRD42022334319).

## Introduction

Neck pain represents a prevalent musculoskeletal condition, with epidemiological characteristics indicating substantial incidence and prevalence rates, imposing significant burdens on both individuals and society at large (1). More than 70 percent of the global population experiences neck pain at least once in their lifetime, with 50–85% likely to experience recurrence within 1–5 years of the initial episode (2, 3). Neck pain is a prevalent condition across all age demographics and genders, exhibiting a particularly high prevalence among individuals aged 45 to 54 years and among females (3–5). Non-specific neck pain, characterized by the absence of detectable systemic disease as the underlying cause, constitutes the predominant form among neck pain cases (6, 7). This condition not only hinders individuals’ daily functioning and vocational abilities but can also lead to chronic functional impairment and a reduced quality of life. Neck pain has become a significant public health issue, calling for effective interventional strategies to alleviate its substantial impact on both individual well-being and societal prosperity.

Virtual Reality (VR) technology presents a groundbreaking approach to addressing this issue. VR generates immersive digital environments using head-mounted displays and other devices (8). With advanced head-mounted displays and related peripherals, users are immersed in a multisensory experience that integrates visual, auditory, and haptic feedback (9–11). In recent years, VR applications have experienced significant growth in rehabilitative medicine, showing remarkable effectiveness in pain management protocols. Scientific studies have highlighted VR’s ability to alleviate pain through mechanisms such as attention diversion, emotional modulation, and enhanced motor engagement (12–14).

VR technology has emerged as a pioneering non-pharmacological intervention, increasingly integrated into neck pain rehabilitation protocols, showing particularly promising potential for pain alleviation and enhancing cervical mobility (15, 16). Although several systematic reviews have examined the benefits of VR for neck pain, they may have inadvertently overlooked the nuanced short-term and long-term efficacy of VR in modulating neck pain (17, 18). Furthermore, these studies may face the risk of false-positive outcomes due to multiple testing and the limitations of available data. With the rapid advancement of technological capabilities and rising service demands, there has been a corresponding increase in publications in this field. Consequently, there is an urgent need to update systematic reviews and meta-analyses, which will provide substantial value to both clinical practitioners and research investigators.

The objective of this study was to conduct a thorough assessment of the effectiveness of virtual reality rehabilitation for patients suffering from neck pain. Additionally, the study implemented trial sequential analysis (TSA) to verify the statistical reliability and methodological robustness of the meta-analytic findings, thereby enhancing the validity and scientific rigor of the derived conclusions.

## Methods

### Protocol and registration

This systematic review adhered rigorously to the Preferred Reporting Items for Systematic Reviews and Meta-Analyses (PRISMA) guidelines to ensure thorough methodological rigor and comprehensive reporting (19). The study protocol was registered with the international open-access Prospective Register of Systematic Reviews (PROSPERO, number: CRD42022334319).

### Literature search

An extensive literature search was performed using databases including PubMed, Embase, Web of Science, Google Scholar, and the Cochrane Library, covering publications from their inception until October 1, 2024. The aim was to identify all relevant randomized controlled trials (RCTs) examining the use of virtual reality-based rehabilitation for neck pain. Search terms combined both indexed terms and medical subject headings (MeSH), such as “virtual reality,” “augmented reality,” “mixed reality,” “neck pain,” and “RCT.” Details of the search strategy applied to each database can be found in Additional file 1.

### Study selection and inclusion criteria

Inclusion criteria were: (1) population: patients experiencing neck pain; (2) intervention: implementation of virtual reality-based rehabilitation; (3) comparison: other interventions (e.g., non-VR approaches, standard care, or no intervention); (4) outcomes: evaluation using the visual analogue scale (VAS) and neck disability index (NDI); (5) study design: exclusively RCTs.

Exclusion criteria encompassed: (1) studies lacking sufficient data; (3) studies without accessible full-text articles.

### Data extraction

Data extraction was meticulously performed by two independent reviewers utilizing a piloted, standardized extraction form. Discrepancies between reviewers were resolved through consensus. Extracted data from each study included: (1) study characteristics such as authorship details, sample size, participant age, and specifics of the intervention versus comparator; (2) outcomes including VAS scores, NDI scores, and range of motion.

### Quality assessment

The assessment of quality and potential bias was conducted using the Cochrane Risk of Bias Tool (20). This evaluation framework considers seven essential domains to determine methodological quality and bias risk: random sequence generation, allocation concealment, blinding of participants and personnel, blinding of outcome assessment, incomplete outcome data, selective reporting, and other biases. Each bias domain was classified as high, unclear, or low risk. Risk of bias graphs were generated using RevMan software (version 5.3).

The Grading of Recommendations Assessment, Development, and Evaluation (GRADE) approach was applied to systematically appraise the methodological quality and reliability of the evidence (21). The evidence quality was categorized into four levels: high, moderate, low, and very low, based on a thorough evaluation of several critical domains. Disparities between the reviewers were deliberated, and if unresolved, a third reviewer was consulted until consensus was achieved.

### Data synthesis

Meta-analyses were conducted using RevMan 5.3 software. Continuous data were evaluated using mean differences with 95% confidence intervals (CIs). For studies with consistent units, the weighted mean difference (WMD) with its 95% CI was preferred; however, when unit inconsistency or significant variability was present, the standardized mean difference (SMD) was applied. Statistical significance was determined at *p* < 0.05, recorded for each outcome measure. If the study results encompassed more than ten articles, a funnel plot should be constructed to evaluate the potential for publication bias. A sensitivity analysis was performed by excluding individual studies to evaluate their impact on overall conclusions, thus affirming result stability.

To minimize the risk of false-positive outcomes due to multiple testing and the constraints of available data, TSA was implemented using TSA Viewer version 0.9.5.10 Beta. This analysis aimed to evaluate the robustness of cumulative evidence related to primary outcomes. We adjusted the required information size (RIS) for our meta-analysis and established trial sequential monitoring boundaries (TSMB) to gauge the reliability and definitiveness of the evidence presented (22). The interaction between the TSA boundary and the cumulative Z-curve was thoroughly examined after calculating sequential monitoring and necessary information size thresholds. If the cumulative Z-curve crossed a trial sequential monitoring boundary, the evidence was considered sufficiently robust to reject the null hypothesis under the prespecified TSA assumptions while accounting for the risk of random error. Exceeding a futility boundary suggested that the prespecified intervention effect was improbable. If neither a monitoring boundary nor a futility boundary was crossed and the required information size had not been reached, the available evidence was deemed inconclusive. Importantly, crossing a monitoring boundary was not interpreted as establishing the clinical importance of the observed effect, exclude systematic bias or unexplained heterogeneity, or suggest that additional research was unnecessary. TSA calculations were performed with a type-1 error α set at 5% and a predefined power (1−β) of 90%. Additional data were sourced from the average incidence across all included studies or those identified as having a low risk of bias (23, 24).

## Results

### Study selection

An extensive systematic literature search was performed across several key databases, including PubMed, Embase, Web of Science, Google Scholar, and the Cochrane Library, initially yielding 352 articles deemed potentially relevant (Figure 1). After eliminating 54 duplicate records, the screening of 298 remaining titles and abstracts resulted in identifying 44 articles as potentially eligible for further analysis. A comprehensive full-text review of these articles led to the exclusion of 33 studies based on predetermined criteria, including inadequate data availability (*n* = 13), classification as review articles (*n* = 14), and being case reports (*n* = 6). Consequently, this systematic review and meta-analysis incorporated a total of 11 RCTs encompassing 476 participants (15, 16, 25–33).

**Figure 1:**
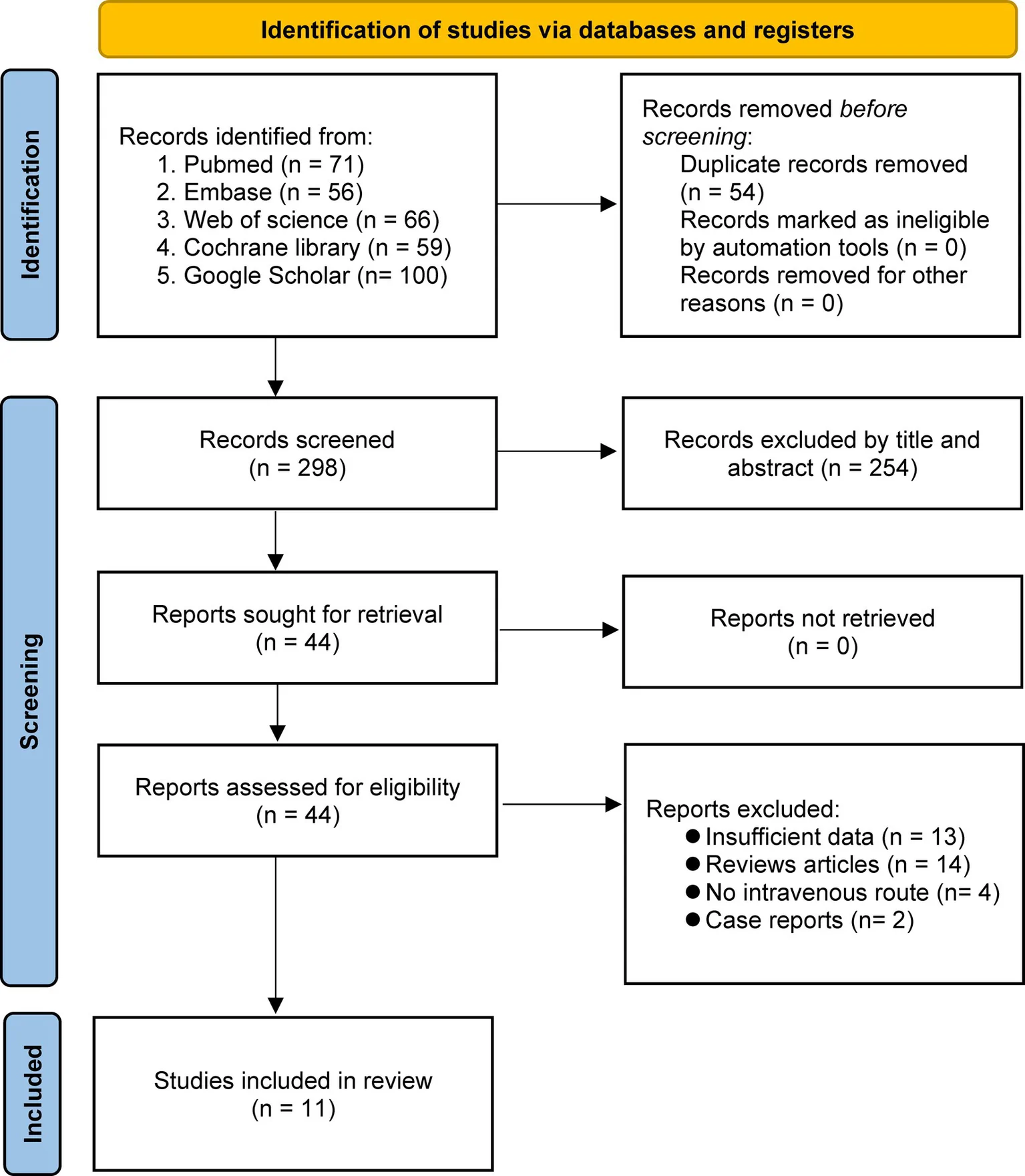
Flowchart for inclusion and exclusion of studies.

### Characteristics of the studies

Table 1 presents the detailed characteristics of the 11 RCTs included in our meta-analysis. One study focused exclusively on young women aged 18 to 25 years (32). Another study specifically recruited participants who were young fighter and helicopter pilots (33). 7 of the 11 studies included patients who had been experiencing neck pain for a duration exceeding 3 months (15, 16, 25, 26, 29–31).

**Table 1:** Basic characteristics of the included studies.

Author	Country	Participants	N (I/C)	Age (Mean ± SD, Y) (I/C)	Women, % (I/C)	Intervention	Comparator	Follow-up
Rezaei, I et al. (15)	Iran	20–55 years; nontraumatic neck pain≥3 months	21/21	36.19 ± 9.80/31.23 ± 9.49	42.9/52.4	VR therapy	Exercise	5 week postoperatively
Sarig Bahat, H et al. (16)	Australia	≥18 years; neck pain≥3 months; NDI score ≥ 10%.	16/14	40.63 ± 14.18/41.13 ± 12.59	37.5/31.3	KT + VR training	KT training	12 week postoperatively
Guo, Q, H et al. (29)	China	≥18 years; neck pain≥3 months; NDI score ≥ 6; NRS ≥ 3	32/32	35.94 ± 11.02/40.09 ± 11.97	78/69	VR therapy	Exercise	12 week postoperatively
Morales Tejera, David, H et al. (28)	Spain	18–65; non-specific chronic neck pain	22/22	32.72 ± 11.63/26.68 ± 9.21	50.0/54.5	VR therapy	Exercise	12 week postoperatively
Nusser, Marina et al. (26)	Germany	≥18 years; nontraumatic neck pain≥3 months; no pain medication or muscle relaxants for 24 h before the tests	17/18	51.2 ± 8.8/49.8 ± 8.1	53/66	Standard rehabilitation program + VR therapy	Standard rehabilitation program	Immediate
			17/16	51.2 ± 8.8/53.1 ± 5.7	53/69	Standard rehabilitation program + VR therapy	Standard rehabilitation program+general sensorimotor training	Immediate
Battecha, Kadrya H et al. (32)	Egypt	18–25; non-specific chronic neck pain	15/15	21.23 ± 0.83/21.26 ± 0.79	100/100	VR therapy	Exercise	Immediate
Mukherjee, Madhumanti et al. (27)	Indian	≥30 years; subacute and chronic cases of cervical spondylosis without radiculopathy	22/21	55.81 ± 15/54.81 ± 13	36.3/61.9	VR therapy	Exercise	3 day postoperatively
Sarig Bahat, H et al. (25)	Australia	≥18 years; neck pain≥3 months	30/30	48/48	63/70	VR therapy	Laser	12 week postoperatively
Bahat, H. S et al. (33)	Israel	fighter and helicopter pilots; neck pain intensity≥20%	22/23	30 ± 5.8/28 ± 5.1	5/13	VR therapy	exercise	24 week postoperatively
Emedoli, D et al. (30)	Italy	18–70 years; neck pain≥3 months	17/16	49.38 ± 10.53/46.05 ± 10.49	N/N	VR therapy	Exercise	Immediate
Cetin, H et al. (31)	Turkey	18–65 years; neck pain≥6 months; NDI score ≥ 20%	17/17	40.0 ± 11.88/41.94 ± 10.76	70.5/64.7	VR therapy	Exercise	Immediate

VR, virtual reality; VAS, Visual Analogue Scale; NDI, Neck Disability Index.

### Risk of bias assessment

The comprehensive bias risk assessment of the included studies revealed an overall low risk (Figures 2, 3). It is worth noting that one study may have a high risk of bias because only the person in charge of the statistical analysis was blinded (28).

**Figure 2:**
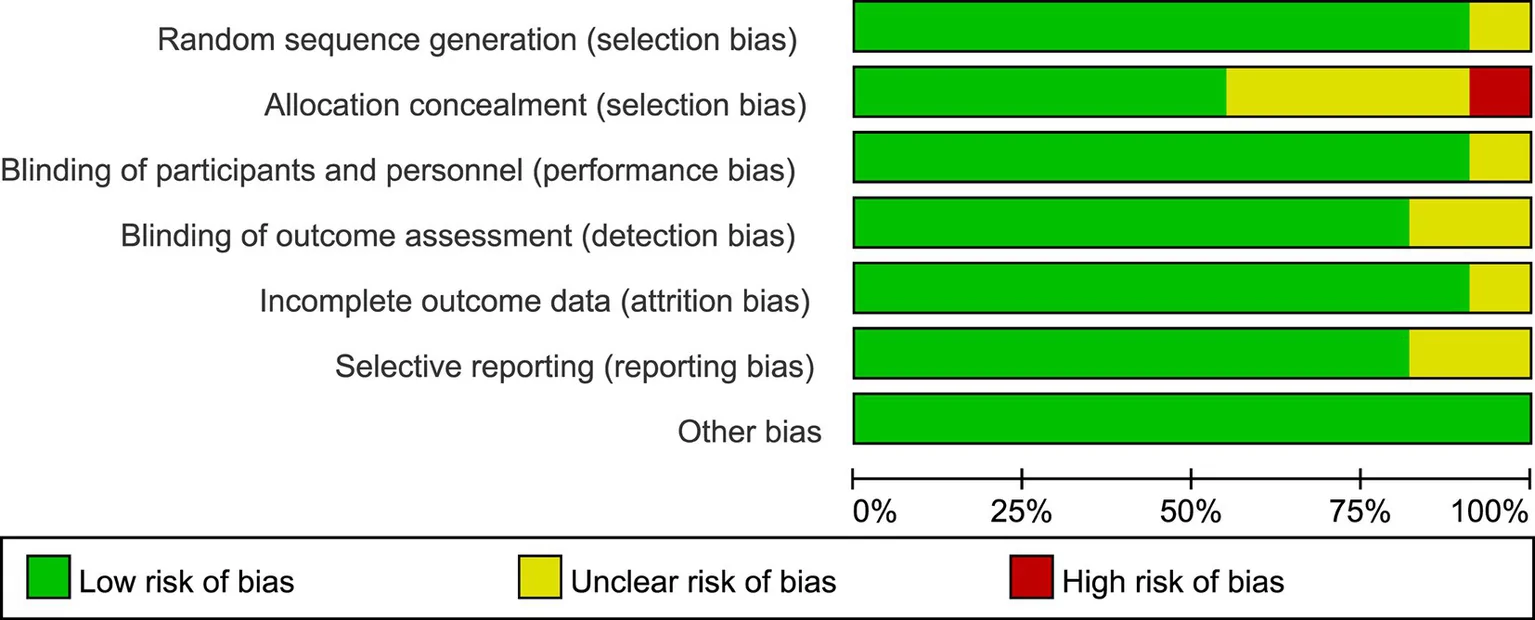
Risk of bias summary.

**Figure 3:**
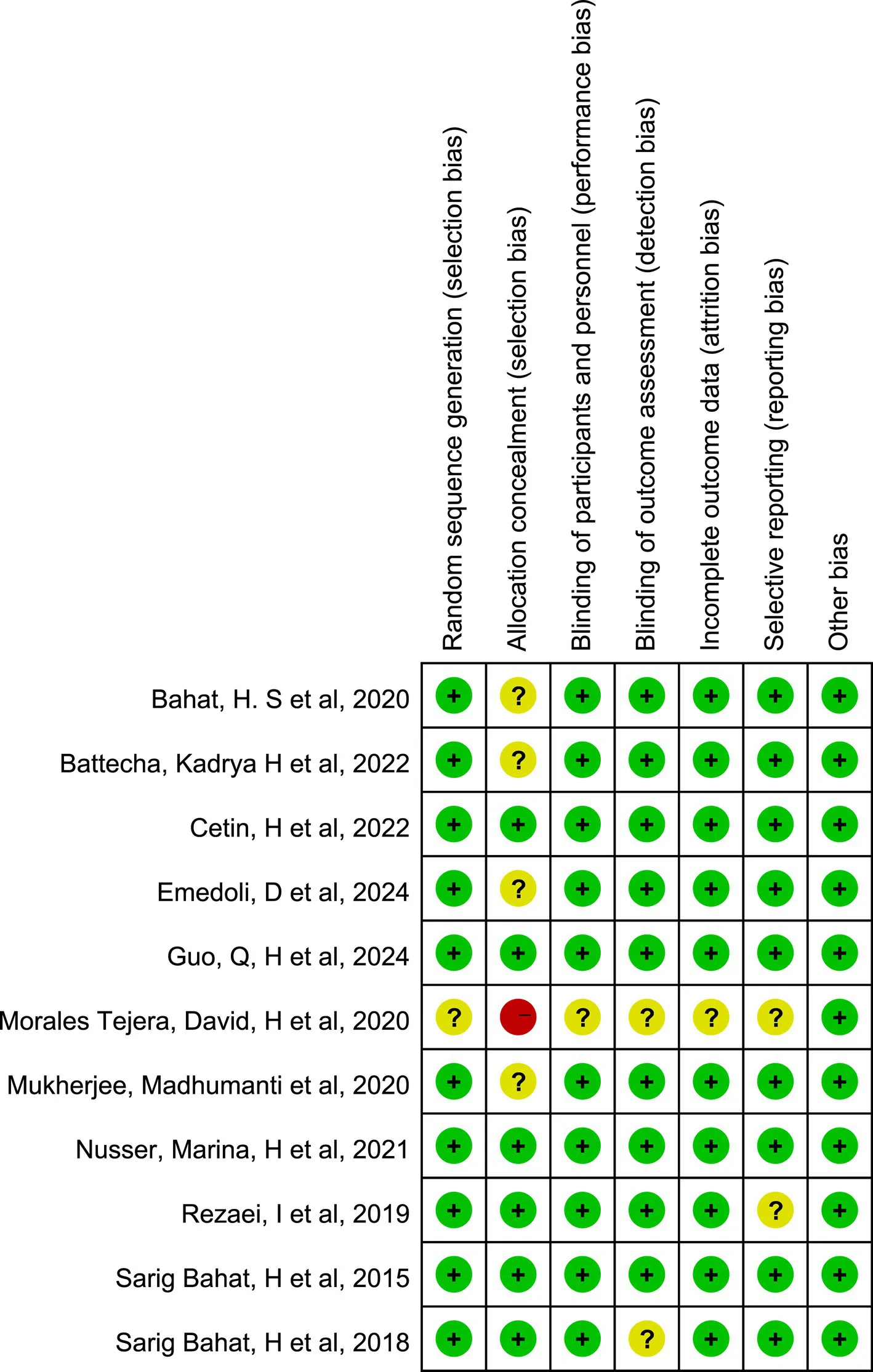
Risk of bias graph.

#### Outcomes

##### Primary outcomes

VAS score within the first 1 week, VAS score from the first 1 week to 6 month postoperatively, NDI score within the first 1 week, NDI score from the first 1 week to 6 month postoperatively.

The meta-analysis identified a significant association between virtual reality-based rehabilitation and a reduction in the VAS scores during the initial week (MD −0.70; 95% CI −1.25 to −0.14, *P* = 0.01, 471 participants, I^2^ = 74%; Figure 4a). Furthermore, patients undergoing this form of rehabilitation experienced a notable decrease in VAS scores extending from the first week to 6 months postoperatively (MD −1.00; 95% CI −1.55 to −0.45; *p* = 0.0004, I^2^ = 51%, 315 participants; Figure 5a). Additionally, virtual reality-based rehabilitation led to a significant reduction in the NDI scores within the first week (MD −3.06; 95% CI −3.84 to −2.29, *p* < 0.0001, I^2^ = 0%, 411 participants; Figure 6a). Regarding the incidence of NDI score changes from the first week to 6 months postoperatively, substantial benefits were observed (MD −3.81; 95% CI −6.45 to −1.18, *p* = 0.005, I^2^ = 84%, 315 participants; Figure 7a). Following harmonization of the outcome scales and reanalysis using mean differences (MDs), the cumulative Z-curves for all primary outcomes crossed both the conventional significance boundaries and the trial sequential monitoring boundaries. Under the prespecified TSA assumptions, these results suggest that the corresponding meta-analytic findings are unlikely to be attributable to random error arising from sparse data or repeated significance testing. The accrued information size, required information size, and TSA-adjusted confidence intervals for each outcome are presented in Figures 4–7. These findings supported the effectiveness of virtual reality-based rehabilitation in successfully reducing both VAS and NDI scores. Concerning publication bias, the funnel plot assessing publication bias for the VAS score within the first week (*p* = 0.338) and the NDI score the first week (*p* = 0.760) demonstrated near symmetry, which suggested a low incidence of publication bias. Given that the number of studies incorporating VAS or NDI score from the first 1 week to 6 month postoperatively was fewer than ten, we failed to conduct a publication bias assessment (Figure 8).

**Figure 4:**
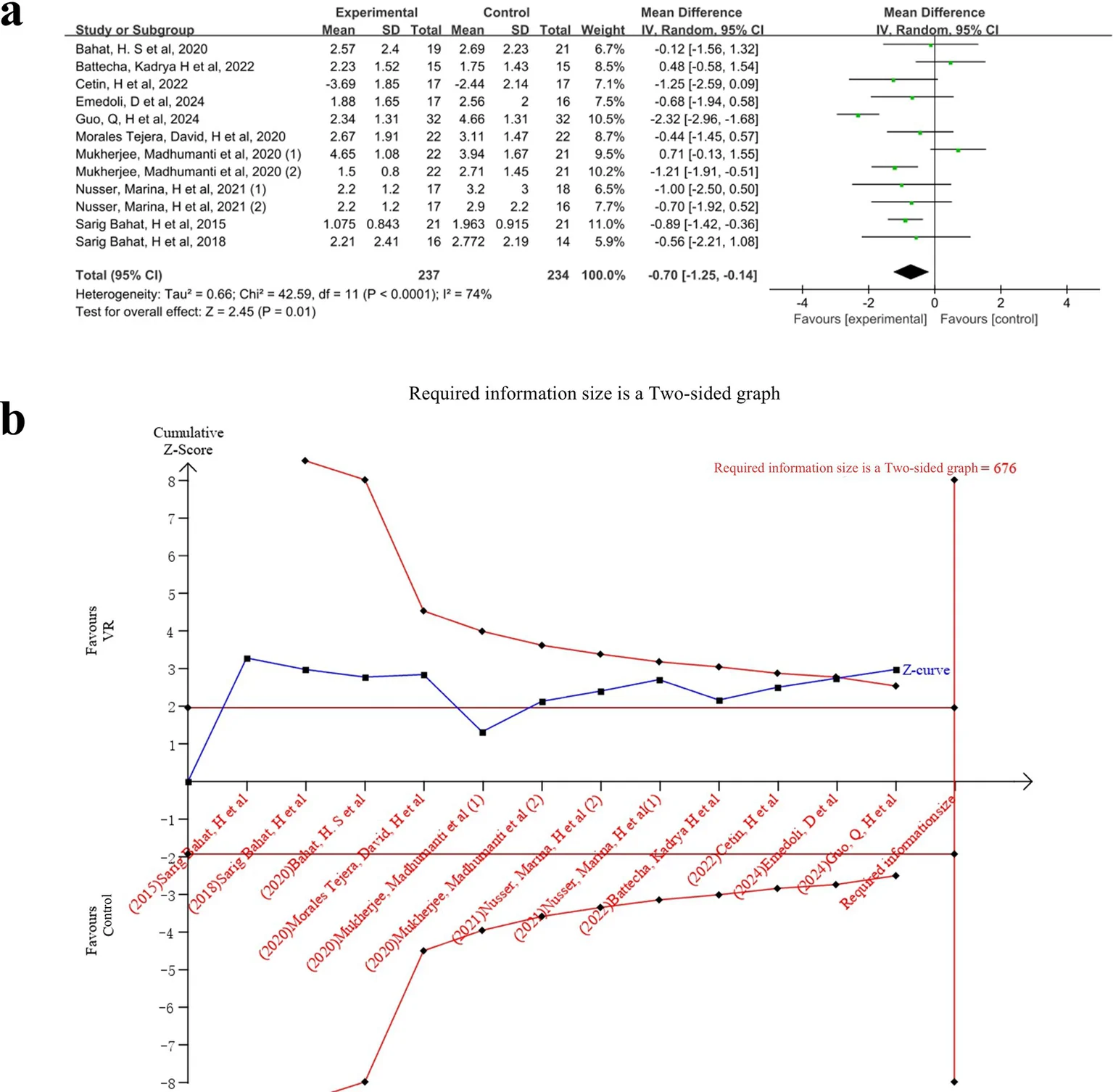
Forest plot showing the association of VR-based rehabilitation with VAS score within the first week. **(a)** Forest plot showing the benefit effect of VR-based rehabilitation on VAS score within the first week. **(b)** Trial sequential analysis supporting the substantial evidence regarding VR-based rehabilitation’s impact on the reduction of VAS score within the first week.

**Figure 5:**
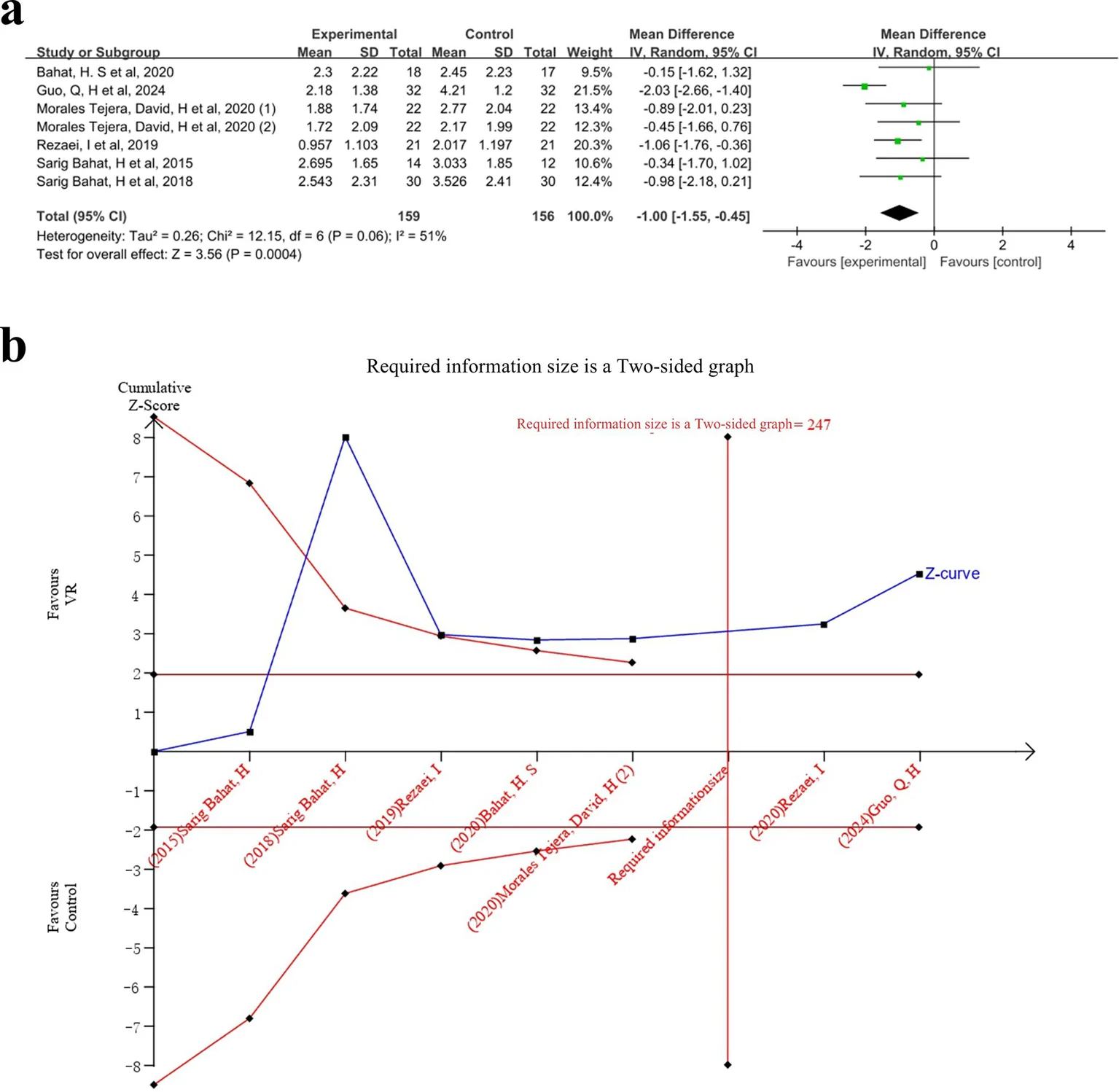
Forest plot showing the association of VR-based rehabilitation with VAS score from the first 1 week to 6 month postoperatively. **(a)** Forest plot showing the benefit effect of VR-based rehabilitation on VAS score from the first 1 week to 6 month postoperatively. **(b)** Trial sequential analysis supporting the substantial evidence regarding VR-based rehabilitation’s impact on the reduction of VAS score from the first 1 week to 6 month postoperatively.

**Figure 6:**
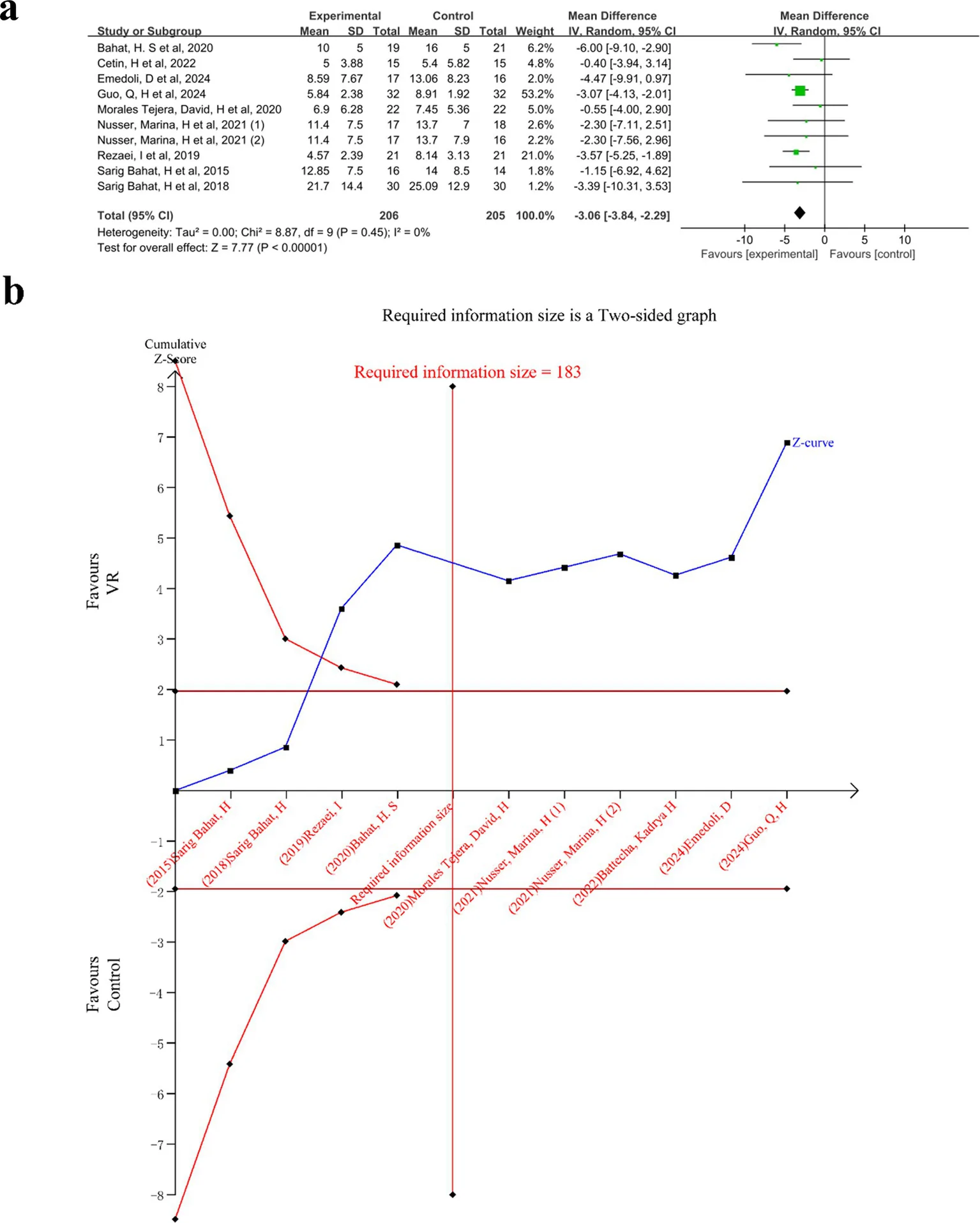
Forest plot showing the association of VR-based rehabilitation with NDI score within the first week. **(a)** Forest plot showing the benefit effect of VR-based rehabilitation on NDI score within the first week. **(b)** Trial sequential analysis supporting the substantial evidence regarding VR-based rehabilitation’s impact on the reduction of NDI score within the first week.

**Figure 7:**
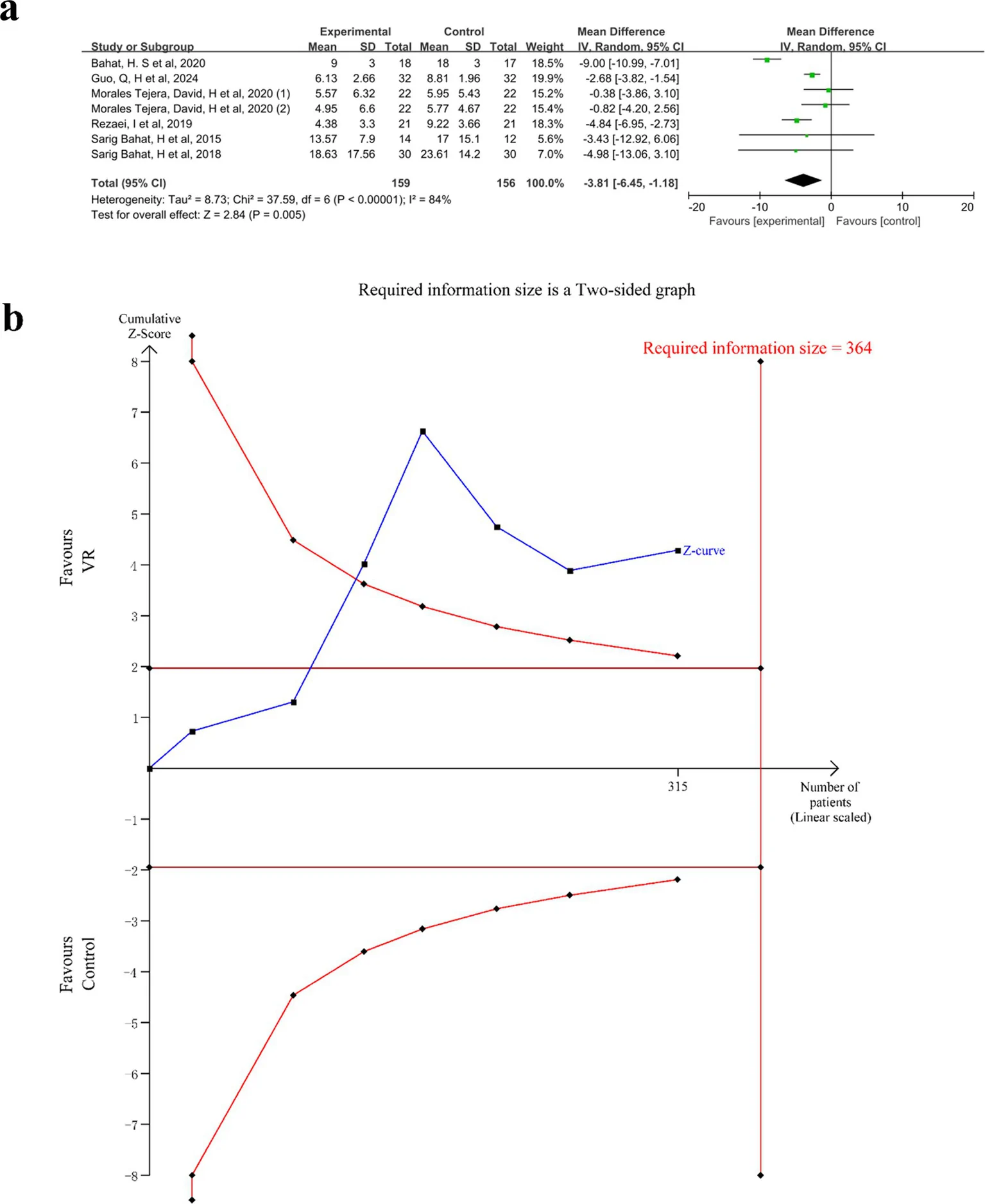
Forest plot showing the association of VR-based rehabilitation with NDI score from the first 1 week to 6 month postoperatively. **(a)** Forest plot showing the benefit effect of VR-based rehabilitation on NDI score from the first 1 week to 6 month postoperatively. **(b)** Trial sequential analysis supporting the substantial evidence regarding VR-based rehabilitation’s impact on the reduction of NDI score from the first 1 week to 6 month postoperatively.

**Figure 8:**
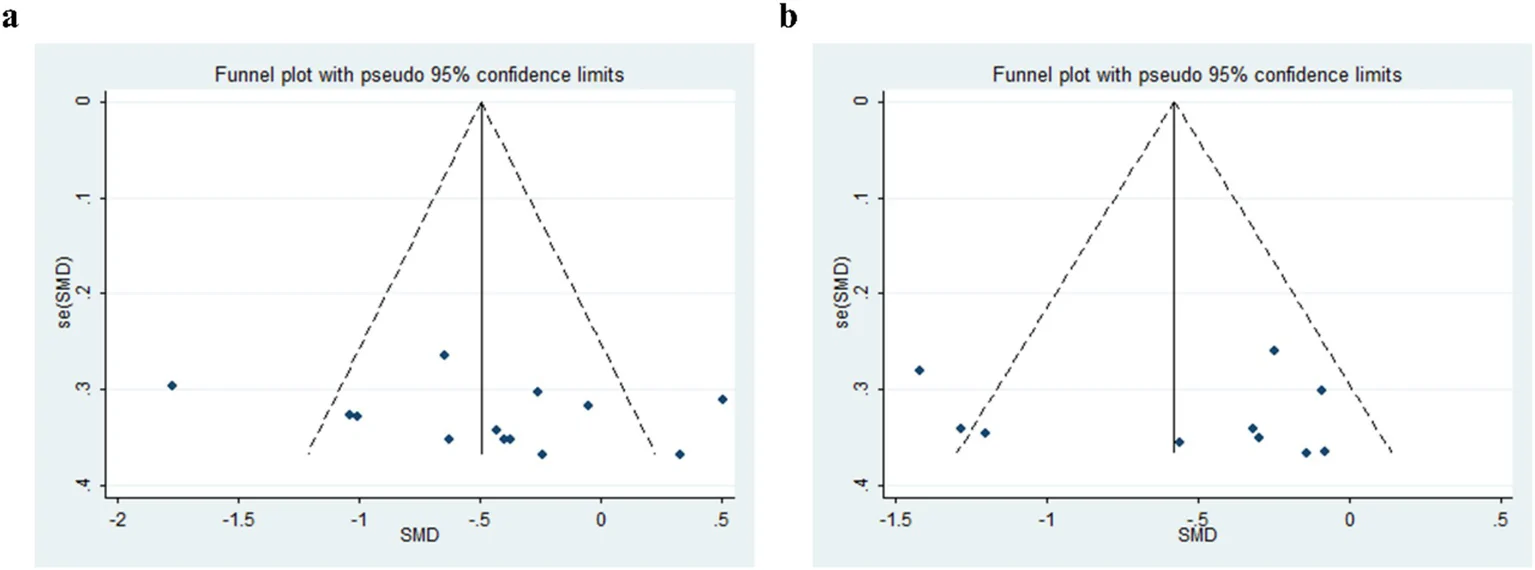
Funnel plot indicating a low risk of bias on the association between VR-based rehabilitation and **(a)** VAS score within the first week (*p* = 0.338); **(b)** NDI score within the first week (*p* = 0.760).

##### The secondary outcomes

Participants who engaged in virtual reality-based rehabilitation exhibited a significant increase in the range of neck flexion (WMD 7.43, 95% CI 4.68 to 10.19, *p* < 0.0001, I^2^ = 0%, 295 participants), neck extension (WMD 6.83, 95% CI 3.82 to 9.84, *p* < 0.0001, I^2^ = 8%, 295 participants), and left neck rotation (WMD 6.38, 95% CI 3.18 to 9.58, *p* < 0.0001, I^2^ = 5%, 265 participants) within the first week when compared to the control group. Furthermore, virtual reality-based rehabilitation was linked to an increased range of neck extension from the first week to 3 months postoperatively (WMD 7.78; 95% CI 0.32 to 15.23, *p* = 0.04, I^2^ = 77%, 197 participants). However, no statistically significant differences were observed in right neck rotation within the first week (WMD 1.52; 95% CI −3.36 to 6.4, *p* = 0.54, I^2^ = 59%, 265 participants), nor in neck flexion (WMD 4.53; 95% CI −5.39 to 14.45, *p* = 0.37, I^2^ = 87%, 197 participants), neck extension (WMD 6.11; 95% CI −5.00 to 17.21, *p* = 0.28, I^2^ = 84%, 197 participants), or right neck rotation (WMD 4.28; 95% CI −6.98 to 15.54, *p* = 0.46, I^2^ = 89%, 197 participants) from 1 week to 3 months postoperatively (Table 2).

**Table 2:** Evaluation of virtual reality (VR) therapy for other health outcomes.

Primary outcome or subgroup		Data sets	Participants	Effect estimate	*p* value	I^2^
Flexion	Within 1 week	7	295	7.43 [4.68, 10.19]	*p* < 0.0001	0%
	1 week to 3 month postoperatively	4	197	4.53 [−5.39, 14.45]	*p* = 0.37	87%
Extension	Within 1 week	7	295	6.83 [3.82, 9.84]	*p* < 0.0001	8%
	1 week to 3 month postoperatively	4	197	7.78 [0.32, 15.23]	*p* = 0.04	77%
Left rotation	Within 1 week	6	265	6.38 [3.18, 9.58]	*p* < 0.0001	5%
	1 week to 3 month postoperatively	4	197	6.11 [−5.00, 17.21]	*p* = 0.28	84%
Right rotation	Within 1 week	6	265	1.52 [−3.36, 6.40]	*p* = 0.54	59%
	1 week to 3 month postoperatively	4	197	4.28 [−6.98, 15.54]	*p* = 0.46	89%

### Sensitivity analysis

The sensitivity analysis indicated that the exclusion of any individual study exerted minimal impact on the overall pooled estimates, which enhanced the reliability of the conclusions drawn from the analysis (Additional file 1).

### Certainty of evidence

Table 3 provided a comprehensive summary of the quality of evidence as evaluated through the GRADE methodology. The evidence quality for primary outcomes varied from moderate to high, thereby reinforcing the robustness and dependability of the conclusions (Table 3).

**Table 3:** Quality of the evidence and recommendation strengths.

Certainty assessment							Number of patients		Effect		Certainty	Importance
Number of studies	Study design	Risk of bias	Inconsistency	Indirectness	Imprecision	Other considerations	Intervention	Control	Relative	Absolute (95% CI)		
VAS within 1 week												
12	Randomized trials	Not serious	Serious	Not serious	Not serious	None	237	234	–	MD 0.7 lower (1.25 lower to 0.14 lower)	⨁⨁⨁◯ Moderate	Critical
VAS 1 week to 6 month postoperatively												
7	Randomized trials	Not serious	Serious	Not serious	Not serious	None	159	156	–	MD 1 lower (1.55 lower to 0.45 lower)	⨁⨁⨁◯ Moderate	Critical
NDI within 1 week												
10	Randomized trials	Not serious	Serious	Not serious	Not serious	None	206	205	–	MD 3.06 lower (3.84 lower to 2.29 lower)	⨁⨁⨁◯ Moderate	Critical
NDI 1 week to 6 month postoperatively												
7	Randomized trials	Not serious	Serious	Not serious	Not serious	None	159	156	–	MD 3.81 lower (6.45 lower to 1.18 lower)	⨁⨁⨁◯ Moderate	Critical

## Discussion

In this systematic review and meta-analysis, which included 11 studies with a combined total of 476 patients, findings indicated that virtual reality-based rehabilitation significantly decreased both VAS and NDI scores within the first week, as well as throughout the period extending up to 6 months postoperatively. TSA was applied to evaluate four primary outcomes, revealing that the cumulative data for all primary outcomes exceeded the conventional Z-value threshold of 1.96 and crossed the TSMB. This result suggested that the available data were sufficient to draw reliable conclusions, thereby confirming that the study’s statistical power had achieved its intended level.

The primary outcomes indicated a significant reduction in VAS scores both within the initial week and persisting up to 6 months following treatment. This suggested that virtual reality-based rehabilitation offers immediate analgesic effects, potentially attributable to its capacity to divert attention from pain and modulate emotional responses. The immersive characteristics of virtual reality experienced may engage patients cognitively, thereby diminishing pain perception (34, 35). The observed sustained reduction in pain over a six-month period suggested that virtual reality may contribute to long-term therapeutic benefits, possibly through mechanisms involving neuroplasticity and increased motor system engagement (36). Clinically, these findings highlighted the potential of virtual reality as a promising non-pharmacological intervention for managing chronic neck pain, thereby reducing dependence on medication and mitigating associated side effects. The observed decrease in NDI scores emphasized the functional advantages of virtual reality interventions, as improved NDI scores correlate with an enhanced capacity to perform daily activities. This suggested that virtual reality not only alleviates pain but also aided in the restoration of functional capacity. Such outcomes were particularly important in the context of neck pain, where functional impairments could profoundly affect quality of life and productivity (37).

The secondary outcomes, demonstrating increased ranges of neck flexion, extension, and left rotation, further underscored the rehabilitative potential of virtual reality. These enhancements in range of motion suggested that virtual reality could effectively improve cervical mobility, which was a vital component of comprehensive pain management strategies (38). The interactive and engaging nature of virtual reality exercises may motivate patients to undertake movements that they might otherwise find challenging, thereby facilitating enhanced rehabilitation outcomes.

VR has been extensively employed in the field of neurological rehabilitation for post-stroke motor function recovery (39), spinal cord injury rehabilitation (40), and the treatment of post-traumatic stress disorder (PTSD) (41). VR rehabilitation offers significant advantages in facilitating physical enhancements, such as improved balance and gait, while concurrently bolstering engagement and motivation among individuals suffering from osteoporosis and other musculoskeletal conditions (42). Interestingly, the forms of VR are incredibly varied, ranging from “virtual reality games” (15) to “VR exercises” (28, 29) and “3D game-based rehabilitation environments” (25). This observation underscores the adaptability for doctors to tailor VR experiences to suit the unique requirements and conditions of their patients.

The efficacy of virtual reality in pain management had been substantiated by multiple studies, with consistent evidence endorsing its application across various types of pain (43, 44). However, this study was distinguished by its rigorous application of TSA, which provided more robust validation of the findings. TSA reduced the risk of type I errors and ensured that the observed effects were corroborated by sufficient cumulative evidence. This methodological enhancement not only strengthened the credibility of the conclusions but also set a new standard for future meta-analyses in this domain. Nevertheless, TSA mainly serves to limit random error arising from data sparsity and repeated significance testing; it does not account for systematic bias, unexplained heterogeneity, indirectness, or publication bias. Moreover, statistical robustness does not necessarily imply clinical meaningfulness. Thus, TSA results should be considered together with conventional confidence intervals, predefined thresholds for clinical importance, risk-of-bias assessments, heterogeneity analyses, and GRADE certainty-of-evidence ratings. Additionally, our study offered a thorough evaluation of both short-term and long-term effects, addressing a significant gap in the literature. This comprehensive approach delivered a nuanced understanding of the potential benefits of virtual reality, highlighting its role as a viable alternative to traditional pain management strategies. Despite these strengths, several limitations must be acknowledged. The observed heterogeneity in certain outcome measures, as indicated by the I^2^ values, suggests variability in study designs, participant characteristics, and intervention protocols, which may influence the generalizability of the results. Future studies should aim to standardize intervention protocols to enhance comparability. Additionally, because fewer than ten studies included VAS or NDI scores from the first week to 6 months postoperatively, we were unable to conduct an assessment of publication bias. Having comprehensively reviewed all available literature on virtual reality treatment for neck pain, we plan to update the data on publication bias as more studies become available in the future.

## Conclusion

In conclusion, this study underscores the potential of virtual reality-based rehabilitation as an innovative, non-pharmacological intervention for managing neck pain, highlighting its ability to relieve pain and improve function. The incorporation of TSA into the analysis enhances the robustness of the conclusions, providing compelling evidence for the efficacy of VR interventions. Future research should aim to address the identified limitations and further investigate the long-term sustainability and cost-effectiveness of VR interventions across diverse patient populations, thereby solidifying its role in comprehensive pain management strategies.

## Data Availability

The authors confirm that the data supporting the findings of this study are available within the article. Relevant data can be available by contacting the corresponding author.
