# Supplementary material for: The effectiveness of virtual reality-based rehabilitation in patients with neck pain: a systematic review and meta-analysis with trial sequential analysis of randomized controlled trials
Source: Front Public Health. 2026 Oct 22;14:1933999. doi: 10.3389/fpubh.2026.1933999 (PMC13736623; doi:10.3389/fpubh.2026.1933999)
Supplement: Supplementary file 1 [file Table_1.DOCX]

Supplemental Table 1. The sensitivity analysis of VAS within 1 week revealed that omitting any study had a relatively low influence on the overall combined estimates.

|  |  | VAS within 1 week |  |  |  |
| --- | --- | --- | --- | --- | --- |
| Study |  | Mean difference | p value |  | I^2^ |
| Bahat, H. S et al, 2020 |  | -0.48 [-0.88, -0.09] | 0.02 |  | 75% |
| Battecha, Kadrya H et al, 2022 |  | -0.51 [-0.88, -0.14] | 0.007 |  | 73% |
| Cetin, H et al, 2022 |  | -0.43 [-0.83, -0.03] | 0.03 |  | 76% |
| Emedoli, D et al, 2024 |  | -0.45 [-0.85, -0.06] | 0.03 |  | 76% |
| Guo, Q, H et al, 2024 |  | -0.32 [-0.60, -0.04] | 0.03 |  | 51% |
| Morales Tejera, David, H et al, 2020 |  | -0.46 [-0.86, -0.06] | 0.02 |  | 75% |
| Mukherjee, Madhumanti et al, 2020 |  | -0.49 [-0.86, -0.12] | 0.01 |  | 68% |
| Nusser, Marina, H et al, 2021 |  | -0.45 [-0.89, -0.02] | 0.04 |  | 78% |
| Sarig Bahat, H et al, 2015 |  | -0.40 [-0.78, -0.01] | 0.04 |  | 74% |
| Sarig Bahat, H et al, 2018 |  | -0.46 [-0.86, -0.07] | 0.02 |  | 76% |

Supplemental Table 2. The sensitivity analysis of VAS 1 week to 6 month postoperatively revealed that omitting any study had a relatively low influence on the overall combined estimates.

|  |  | VAS 1 week to 6 month postoperatively | | |  |
| --- | --- | --- | --- | --- | --- |
| Study |  | Mean difference | p value |  | I2 |
| Bahat, H. S et al, 2020 |  | -0.64 [-1.06, -0.21] | 0.004 |  | 67% |
| Guo, Q, H et al, 2024 |  | -0.39 [-0.64, -0.14] | 0.002 |  | 0% |
| Morales Tejera, David, H et al, 2020 |  | -0.64 [-1.19, -0.10] | 0.02 |  | 74% |
| Rezaei, I et al, 2019 |  | -0.50 [-0.95, -0.05] | 0.03 |  | 70% |
| Sarig Bahat, H et al, 2015 |  | -0.61 [-1.05, -0.17] | 0.007 |  | 70% |
| Sarig Bahat, H et al, 2018 |  | -0.58 [-1.06, -0.10] | 0.02 |  | 71% |

Supplemental Table 3. The sensitivity analysis of NDI within 1 week revealed that omitting any study had a relatively low influence on the overall combined estimates.

|  |  | NDI within 1 week |  |  |  |
| --- | --- | --- | --- | --- | --- |
| Study |  | Mean difference | p value |  | I2 |
| Bahat, H. S et al, 2020 |  | -0.50 [-0.84, -0.15] | 0.005 |  | 62% |
| Cetin, H et al, 2022 |  | -0.61 [-0.96, -0.26] | 0.0007 |  | 64% |
| Emedoli, D et al, 2024 |  | -0.56 [-0.93, -0.19] | 0.003 |  | 67% |
| Guo, Q, H et al, 2024 |  | -0.45 [-0.74, -0.16] | 0.002 |  | 44% |
| Morales Tejera, David, H et al, 2020 |  | -0.62 [-0.97, -0.26] | 0.0006 |  | 63% |
| Nusser, Marina, H et al, 2021 |  | -0.62 [-1.03, -0.22] | 0.003 |  | 70% |
| Rezaei, I et al, 2019 |  | -0.49 [-0.82, -0.15] | 0.005 |  | 60% |
| Sarig Bahat, H et al, 2015 |  | -0.60 [-0.96, -0.25] | 0.0009 |  | 65% |
| Sarig Bahat, H et al, 2018 |  | -0.60 [-0.97, -0.23] | 0.001 |  | 65% |

Supplemental Table 4. The sensitivity analysis of NDI 1 week to 6 month postoperatively revealed that omitting any study had a relatively low influence on the overall combined estimates.

|  |  | NDI 1 week to 6 month postoperatively | | |  |
| --- | --- | --- | --- | --- | --- |
| Study |  | Mean difference | p value |  | I2 |
| Bahat, H. S et al, 2020 |  | -0.55 [-0.99, -0.11] | 0.01 |  | 69% |
| Guo, Q, H et al, 2024 |  | -0.78 [-1.48, -0.08] | 0.03 |  | 85% |
| Morales Tejera, David, H et al, 2020 |  | -1.14 [-1.90, -0.38] | 0.003 |  | 85% |
| Rezaei, I et al, 2019 |  | -0.74 [-1.40, -0.08] | 0.03 |  | 85% |
| Sarig Bahat, H et al, 2015 |  | -0.92 [-1.60, -0.24] | 0.008 |  | 86% |
| Sarig Bahat, H et al, 2018 |  | -0.93 [-1.65, -0.22] | 0.01 |  | 86% |

**Search strategy**

**Pubmed**

(((virtual reality[Title/Abstract]) OR (Reality, Virtual[Title/Abstract]) OR (Virtual Reality, Educational[Title/Abstract]) OR (Educational Virtual Realities[Title/Abstract]) OR (Educational Virtual Reality[Title/Abstract]) OR (Reality, Educational Virtual[Title/Abstract]) OR (Virtual Realities, Educational[Title/Abstract]) OR (Virtual Reality, Instructional[Title/Abstract]) OR (Instructional Virtual Realities[Title/Abstract]) OR (Instructional Virtual Reality[Title/Abstract]) OR (Realities, Instructional Virtual[Title/Abstract]) OR (Reality, Instructional Virtual[Title/Abstract]) OR (Virtual Realities, Instructional[Title/Abstract]) OR (augmented reality[Title/Abstract]) OR(Augmented Realities[Title/Abstract]) OR(Realities, Augmented[Title/Abstract]) OR(Reality, Augmented[Title/Abstract]) OR(Mixed Reality[Title/Abstract]) OR(Mixed Realities[Title/Abstract]) OR (Realities, Mixed[Title/Abstract]) OR (Reality, Mixed[Title/Abstract]) OR (mixed reality[Title/Abstract])) AND ((neck pain[Title/Abstract]) OR (Neck Pains[Title/Abstract]) OR (Pain, Neck[Title/Abstract]) OR (Pains, Neck[Title/Abstract]) OR (Neckache[Title/Abstract]) OR (Neckaches[Title/Abstract]) OR (Cervical Pain[Title/Abstract]) OR (Cervical Pains[Title/Abstract]) OR (Pain, Cervical[Title/Abstract]) OR (Pains, Cervical[Title/Abstract]) OR (Cervicalgia[Title/Abstract]) OR (Cervicalgias[Title/Abstract]) OR (Cervicodynia[Title/Abstract]) OR (Cervicodynias[Title/Abstract]) OR (Neck Ache[Title/Abstract]) OR (Ache, Neck[Title/Abstract]) OR (Aches, Neck[Title/Abstract]) OR (Neck Aches[Title/Abstract]) OR (Anterior Cervical Pain[Title/Abstract]) OR (Anterior Cervical Pains[Title/Abstract]) OR (Cervical Pain, Anterior[Title/Abstract]) OR (Cervical Pains, Anterior[Title/Abstract]) OR (Pain, Anterior Cervical[Title/Abstract]) OR (Pains, Anterior Cervical[Title/Abstract]) OR (Anterior Neck Pain[Title/Abstract]) OR (Anterior Neck Pains[Title/Abstract]) OR (Neck Pain, Anterior[Title/Abstract]) OR (Neck Pains, Anterior[Title/Abstract]) OR (Pain, Anterior Neck[Title/Abstract]) OR (Pains, Anterior Neck[Title/Abstract]) OR (Posterior Cervical Pain[Title/Abstract]) OR (Cervical Pain, Posterior[Title/Abstract]) OR (Cervical Pains, Posterior[Title/Abstract]) OR (Pain, Posterior Cervical[Title/Abstract]) OR (Pains, Posterior Cervical[Title/Abstract]) OR (Posterior Cervical Pains[Title/Abstract]) OR (Posterior Neck Pain[Title/Abstract]) OR (Neck Pain, Posterior[Title/Abstract]) OR (Neck Pains, Posterior[Title/Abstract]) OR (Pain, Posterior Neck[Title/Abstract]) OR (Pains, Posterior Neck[Title/Abstract]) OR (Posterior Neck Pains[Title/Abstract]))) AND ((RCT[Title/Abstract]) OR (RCTs[Title/Abstract]) OR (Randomized Controlled Trials as Topic[Title/Abstract]) OR (Clinical Trials, Randomized[Title/Abstract]) OR (Trials, Randomized Clinical[Title/Abstract]) OR (Controlled Clinical Trials, Randomized[Title/Abstract]) OR (Randomized Controlled Trials[Title/Abstract]) OR (Randomized Controlled Trial[Title/Abstract]))

**WOS**

1: TI=(virtual reality OR Reality, Virtual OR Virtual Reality, Educational OR Educational Virtual Realities OR Educational Virtual Reality OR Reality, Educational Virtual OR Virtual Realities, Educational OR Virtual Reality, Instructional OR Instructional Virtual Realities OR Instructional Virtual Reality OR Realities, Instructional Virtual OR Reality, Instructional Virtual OR Virtual Realities, Instructional OR augmented reality OR Augmented Realities OR Realities, Augmented OR Reality, Augmented OR Mixed Reality OR Mixed Realities OR Realities, Mixed OR Reality, Mixed OR mixed reality ) and Preprint Citation Index (Exclude – Database)

2: TI=(neck pain OR Neck Pains OR Pain, Neck OR Pains, Neck OR Neckache OR Neckaches OR Cervical Pain OR Cervical Pains OR Pain, Cervical OR Pains, Cervical OR Cervicalgia OR Cervicalgias OR Cervicodynia OR Cervicodynias OR Neck Ache OR Ache, Neck OR Aches, Neck OR Neck Aches OR Anterior Cervical Pain OR Anterior Cervical Pains OR Cervical Pain, Anterior OR Cervical Pains, Anterior OR Pain, Anterior Cervical OR Pains, Anterior Cervical OR Anterior Neck Pain OR Anterior Neck Pains OR Neck Pain, Anterior OR Neck Pains, Anterior OR Pain, Anterior Neck OR Pains, Anterior Neck OR Posterior Cervical Pain OR Cervical Pain, Posterior OR Cervical Pains, Posterior OR Pain, Posterior Cervical OR Pains, Posterior Cervical OR Posterior Cervical Pains OR Posterior Neck Pain OR Neck Pain, Posterior OR Neck Pains, Posterior OR Pain, Posterior Neck OR Pains, Posterior Neck OR Posterior Neck Pains ) and Preprint Citation Index (Exclude – Database)

3: TI=(RCT OR RCTs OR Randomized Controlled Trials as Topic OR Clinical Trials, Randomized OR Trials, Randomized Clinical OR Controlled Clinical Trials, Randomized OR Randomized Controlled Trials OR Randomized Controlled Trial) and Preprint Citation Index (Exclude – Database)

4: #1 AND #2 AND #3

**Embase**

#1 AND #2 AND #3

#3 'rct':ti,ab,kw OR 'rcts':ti,ab,kw OR 'randomized controlled trials as topic':ti,ab,kw OR 'clinical trials, randomized':ti,ab,kw OR 'trials, randomized clinical':ti,ab,kw OR 'controlled clinical trials, randomized':ti,ab,kw OR 'randomized controlled trials':ti,ab,kw OR 'randomized controlled trial':ti,ab,kw

#2 'neck pain':ti,ab,kw OR 'neck pains':ti,ab,kw OR 'pain, neck':ti,ab,kw OR 'pains, neck':ti,ab,kw OR 'neckache':ti,ab,kw OR 'neckaches':ti,ab,kw OR 'cervical pain':ti,ab,kw OR 'cervical pains':ti,ab,kw OR 'pain, cervical':ti,ab,kw OR 'pains, cervical':ti,ab,kw OR 'cervicalgia':ti,ab,kw OR 'cervicalgias':ti,ab,kw OR 'cervicodynia':ti,ab,kw OR 'cervicodynias':ti,ab,kw OR 'neck ache':ti,ab,kw OR 'ache, neck':ti,ab,kw OR 'aches, neck':ti,ab,kw OR 'neck aches':ti,ab,kw OR 'anterior cervical pain':ti,ab,kw OR 'anterior cervical pains':ti,ab,kw OR 'cervical pain, anterior':ti,ab,kw OR 'cervical pains, anterior':ti,ab,kw OR 'pain, anterior cervical':ti,ab,kw OR 'pains, anterior cervical':ti,ab,kw OR 'anterior neck pain':ti,ab,kw OR 'anterior neck pains':ti,ab,kw OR 'neck pain, anterior':ti,ab,kw OR 'neck pains, anterior':ti,ab,kw OR 'pain, anterior neck':ti,ab,kw OR 'pains, anterior neck':ti,ab,kw OR 'posterior cervical pain':ti,ab,kw OR 'cervical pain, posterior':ti,ab,kw OR 'cervical pains, posterior':ti,ab,kw OR 'pain, posterior cervical':ti,ab,kw OR 'pains, posterior cervical':ti,ab,kw OR 'posterior cervical pains':ti,ab,kw OR 'posterior neck pain':ti,ab,kw OR 'neck pain, posterior':ti,ab,kw OR 'neck pains, posterior':ti,ab,kw OR 'pain, posterior neck':ti,ab,kw OR 'pains, posterior neck':ti,ab,kw OR 'posterior neck pains':ti,ab,kw

#1 'virtual reality':ti,ab,kw OR 'reality, virtual':ti,ab,kw OR 'virtual reality, educational':ti,ab,kw OR 'educational virtual realities':ti,ab,kw OR 'educational virtual reality':ti,ab,kw OR 'reality, educational virtual':ti,ab,kw OR 'virtual realities, educational':ti,ab,kw OR 'virtual reality, instructional':ti,ab,kw OR 'instructional virtual realities':ti,ab,kw OR 'instructional virtual reality':ti,ab,kw OR 'realities, instructional virtual':ti,ab,kw OR 'reality, instructional virtual':ti,ab,kw OR 'virtual realities, instructional':ti,ab,kw OR 'augmented reality':ti,ab,kw OR 'augmented realities':ti,ab,kw OR 'realities, augmented':ti,ab,kw OR 'reality, augmented':ti,ab,kw OR 'mixed realities':ti,ab,kw OR 'realities, mixed':ti,ab,kw OR 'reality, mixed':ti,ab,kw OR 'mixed reality':ti,ab,kw

**COC**

#1	(virtual reality):ti,ab,kw OR (Reality, Virtual):ti,ab,kw OR (Virtual Reality, Educational):ti,ab,kw OR (Educational Virtual Realities):ti,ab,kw OR (Educational Virtual Reality):ti,ab,kw OR (Reality, Educational Virtual):ti,ab,kw OR (Virtual Realities, Educational):ti,ab,kw OR (Virtual Reality, Instructional):ti,ab,kw OR (Instructional Virtual Realities):ti,ab,kw OR (Instructional Virtual Reality):ti,ab,kw OR (Realities, Instructional Virtual):ti,ab,kw OR (Reality, Instructional Virtual):ti,ab,kw OR (Virtual Realities, Instructional):ti,ab,kw OR (augmented reality):ti,ab,kw OR (Augmented Realities):ti,ab,kw OR (Realities, Augmented):ti,ab,kw OR (Reality, Augmented):ti,ab,kw OR (Mixed Reality):ti,ab,kw OR (Mixed Realities):ti,ab,kw OR (Realities, Mixed):ti,ab,kw OR (Reality, Mixed):ti,ab,kw OR (mixed reality):ti,ab,kw

#2	(neck pain):ti,ab,kw OR (Neck Pains):ti,ab,kw OR (Pain, Neck):ti,ab,kw OR (Pains, Neck):ti,ab,kw OR (Neckache):ti,ab,kw OR (Neckaches):ti,ab,kw OR (Cervical Pain):ti,ab,kw OR (Cervical Pains):ti,ab,kw OR (Pain, Cervical):ti,ab,kw OR (Pains, Cervical):ti,ab,kw OR (Cervicalgia):ti,ab,kw OR (Cervicalgias):ti,ab,kw OR (Cervicodynia):ti,ab,kw OR (Cervicodynias):ti,ab,kw OR (Neck Ache):ti,ab,kw OR (Ache, Neck):ti,ab,kw OR (Aches, Neck):ti,ab,kw OR (Neck Aches):ti,ab,kw OR (Anterior Cervical Pain):ti,ab,kw OR (Anterior Cervical Pains):ti,ab,kw OR (Cervical Pain, Anterior):ti,ab,kw OR (Cervical Pains, Anterior):ti,ab,kw OR (Pain, Anterior Cervical):ti,ab,kw OR (Pains, Anterior Cervical):ti,ab,kw OR (Anterior Neck Pain):ti,ab,kw OR (Anterior Neck Pains):ti,ab,kw OR (Neck Pain, Anterior):ti,ab,kw OR (Neck Pains, Anterior):ti,ab,kw OR (Pain, Anterior Neck):ti,ab,kw OR (Pains, Anterior Neck):ti,ab,kw OR (Posterior Cervical Pain):ti,ab,kw OR (Cervical Pain, Posterior):ti,ab,kw OR (Cervical Pains, Posterior):ti,ab,kw OR (Pain, Posterior Cervical):ti,ab,kw OR (Pains, Posterior Cervical):ti,ab,kw OR (Posterior Cervical Pains):ti,ab,kw OR (Posterior Neck Pain):ti,ab,kw OR (Neck Pain, Posterior):ti,ab,kw OR (Neck Pains, Posterior):ti,ab,kw OR (Pain, Posterior Neck):ti,ab,kw OR (Pains, Posterior Neck):ti,ab,kw OR (Posterior Neck Pains):ti,ab,kw

#3	(RCT):ti,ab,kw OR (RCTs):ti,ab,kw OR (Randomized Controlled Trials as Topic):ti,ab,kw OR (Clinical Trials, Randomized):ti,ab,kw OR (Trials, Randomized Clinical):ti,ab,kw OR (Controlled Clinical Trials, Randomized):ti,ab,kw OR (Randomized Controlled Trials):ti,ab,kw OR (Randomized Controlled Trial):ti,ab,kw	863642

#4	#1 AND #2 AND #3
